# Juvenile hormone suppresses aggregation behavior through influencing antennal gene expression in locusts

**DOI:** 10.1371/journal.pgen.1008762

**Published:** 2020-04-29

**Authors:** Wei Guo, Juan Song, Pengcheng Yang, Xiangyong Chen, Dafeng Chen, Dani Ren, Le Kang, Xianhui Wang

**Affiliations:** 1 State Key Laboratory of Integrated Management of Pest Insects and Rodents, Institute of Zoology, Chinese Academy of Sciences, Beijing, China; 2 CAS Center for Excellence in Biotic Interactions, University of Chinese Academy of Sciences, Beijing, China; 3 Beijing Institutes of Life Science, Chinese Academy of Sciences, Beijing, China; Howard Hughes Medical Institute, UNITED STATES

## Abstract

Animals often exhibit dramatically behavioral plasticity depending on their internal physiological state, yet little is known about the underlying molecular mechanisms. The migratory locust, *Locusta migratoria*, provides an excellent model for addressing these questions because of their famous phase polyphenism involving remarkably behavioral plasticity between gregarious and solitarious phases. Here, we report that a major insect hormone, juvenile hormone, is involved in the regulation of this behavioral plasticity related to phase change by influencing the expression levels of olfactory-related genes in the migratory locust. We found that the treatment of juvenile hormone analog, methoprene, can significantly shift the olfactory responses of gregarious nymphs from attraction to repulsion to the volatiles released by gregarious nymphs. In contrast, the repulsion behavior of solitarious nymphs significantly decreased when they were treated with precocene or injected with double-stranded RNA of *JHAMT*, a juvenile hormone acid O-methyltransferase. Further, JH receptor *Met* or JH-response gene *Kr-h1* knockdown phenocopied the JH-deprivation effects on olfactory behavior. RNA-seq analysis identified 122 differentially expressed genes in antennae after methoprene application on gregarious nymphs. Interestingly, several olfactory-related genes were especially enriched, including *takeout* (*TO*) and *chemosensory protein* (*CSP*) which have key roles in behavioral phase change of locusts. Furthermore, methoprene application and *Met* or *Kr-h1* knockdown resulted in simultaneous changes of both *TO1* and *CSP3* expression to reverse pattern, which mediated the transition between repulsion and attraction responses to gregarious volatiles. Our results suggest the regulatory roles of a pleiotropic hormone in locust behavioral plasticity through modulating gene expression in the peripheral olfactory system.

## Author summary

A behavioral change from shy solitarious individuals to highly social gregarious individuals is critical to the formation of disastrous swarms of locusts. However, the underlying molecular mechanism of behavioral plasticity regulated by hormones is still largely unknown. Here, we investigated the effect of juvenile hormone (JH) on the behavioral transition in fourth-instar gregarious and solitarious locusts. We found that JH induced the behavioral shift of the gregarious locust from attraction to repulsion to the volatiles of gregarious locusts. The solitarious locust significantly decreased repulsion behavior after deprivation of JH by precocene or knockdown of *JHAMT*, a key enzyme to synthesize JH. JH application on gregarious locusts caused significant expression alteration of genes, especially the olfactory genes *TO* and *CSP* in the antennae. We further demonstrated that the JH signaling pathway suppressed aggregation behavior in gregarious locusts by increasing *TO1* expression and decreasing *CSP3* expression at the same time. Our results suggested that internal physiological factors can directly modulate periphery olfactory system to produce behavioral plasticity.

## Introduction

Insects generally express behavioral plasticity to adapt environmental changes by integrating precisely internal and external chemical cues. A number of studies have suggested the coordinated roles of juvenile hormone (JH), which is secreted by the corpora allata and as one of the most important insect hormones, on behavioral plasticity in several insect species **[1–3]**. Recently, JH have been demonstrated to mediate long lasting genomic modifications or signal transduction cascades associated with either the central **[4]** or the peripheral nervous systems **[5]**, which can lead to overt behavioral changes. However, the detailed molecular mechanisms underlying the action of JH on behavioral plasticity remain largely unknown.

The migratory locust, *Locusta migratoria*, displays a remarkable density-dependent behavioral plasticity involving swarm formation and large-scale migration **[6–8]**. Gregarious locusts in high density actively aggregate; however, solitarious locusts at low density are shy and actively avoid each other. This attraction/repulsion behavior of locusts has been demonstrated to be induced exclusively by conspecific olfactory cues **[9–13]**. The shift between attractive and repulsive responses to olfactory cues can occur rapidly within several hours with changes in population density **[14, 15]**. Our previous studies have revealed that the detailed regulatory mechanisms underlying phase-dependent olfactory-mediated behavioral plasticity occurred at the central nervous system (CNS), including several monoamine signaling pathways **[6]**. For example, the higher concentration of dopamine and expression levels of key genes in the dopamine metabolic pathway induce olfactory attraction in gregarious locusts **[16, 17]**. The role of dopamine in olfactory attraction is mediated by Dop1–miR-9a–AC2 circuit in the Kenyon cells of mushroom body **[10]**. In addition, the invertebrate-specific octopamine-OARa and tyramine-TAR signaling pathways are also involved in the regulation of attractive and repulsive behavior probably in the primary olfactory center of the *L*. *migratoria* **[14]**. Besides, at the peripheral nervous system, significant phase differences were also found in the responses of antennal olfactory receptor neurons to odours **[18]**. Several antennal tissue-enriched gene categories, including *CSP* and *takeout*, have been confirmed to be involved in the shift of attraction/repulsion behavior **[15]**. However, an understanding of global regulatory factors remains largely unknown at the peripheral olfactory system level.

JH has been proposed to be involved in shaping phase polyphenism in locusts **[8, 19]**. Endogenous JH titres and expression levels of several gene categories related to the JH pathway display significant phase-related differences in *L*. *migratoria* **[7, 20]**. Pheromone release and olfactory responsiveness appears to be under the control of JH in another locust species *Schistocerca gregaria* **[21, 22]**. Several studies proposed that JH may have a possible role in the olfactory sensitivity in *S*. *gregaria* **[23, 24]**. Therefore, we hypothesized that JH may be involved in the regulation of phase-related olfactory-mediated behavioral plasticity in locusts.

In this study, we investigated the effect of JH on behavioral responses to the volatiles from gregarious nymphs of *L*. *migratoria* through pharmacological intervention and RNAi. Subsequent antennae RNA-seq analysis was performed to identify phase- and JH-related gene expression differences and candidate genes involved in the influence of JH on the peripheral olfactory system. Our findings provide insight into the roles of JH in the modulation of locust phase polyphenism.

## Results

### The effects of JH analog on attraction/repulsion behavior

**Fig 1:**
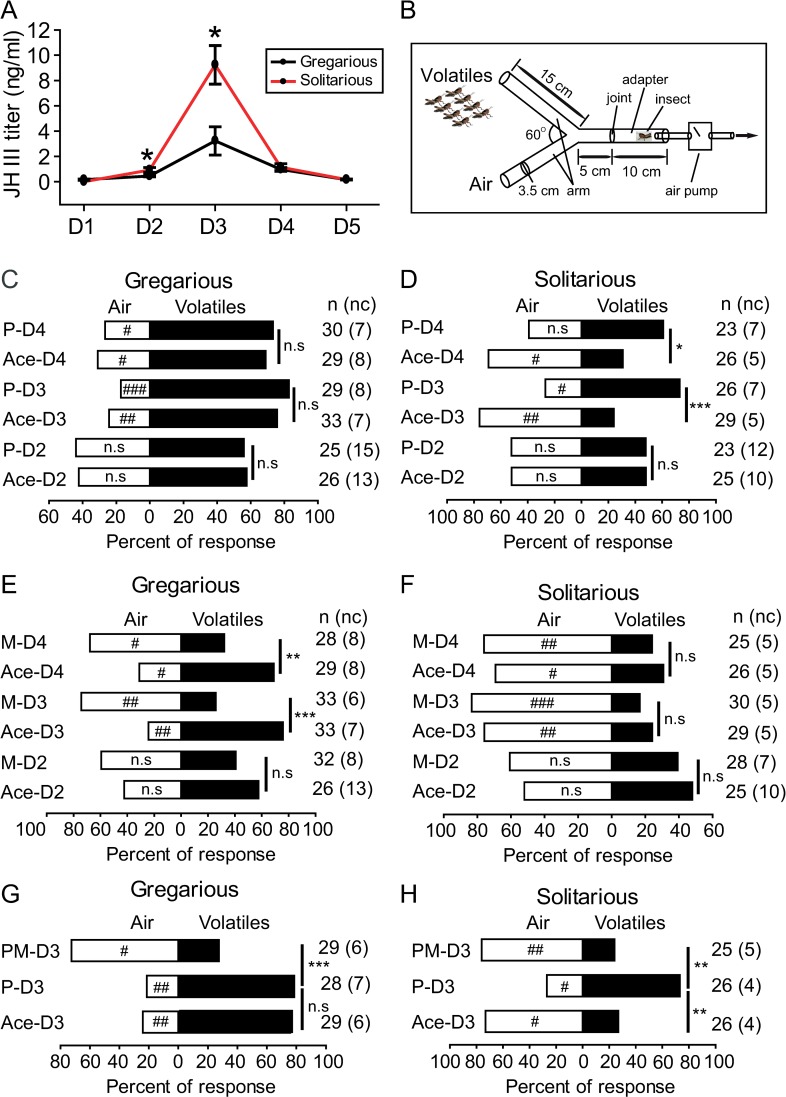
Juvenile hormone titre and its influence on phase-related olfactory behavior in fourth-instar nymphs. (A) JH III titres in hemolymph of gregarious or solitarious nymphs. (B) Schematic diagram of Y-tube olfactometer assay set. Individual nymph was released from Y-tube adapter to observe their choice. (C) Choice behavior of gregarious nymphs at day 2, 3 and 4 (D2,3 and 4) treated with acetone (Ace) or precocene (P) at day 1. (D) Choice behavior of gregarious nymphs at day 2, 3 and 4 treated with acetone or methoprene (M) at day 1. (E) Choice behavior of solitarious nymphs at day 2, 3 and 4 treated with acetone or precocene at day 1. (F) Choice behavior of solitarious nymphs at day 2, 3 and 4 treated with acetone or methoprene at day 1. (G) Choice behavior of gregarious nymphs at day 3 treated with precocene or rescued by methoprene. (H) Choice behavior of solitarious nymphs at day 3 treated with precocene or rescued by methoprene. Volatiles represent volatiles emanating from 30 fourth-instar gregarious nymphs. Significance marks inside white stripe indicate the significance between individuals choosing air side and volatiles side. Marks outside the stripe indicate the significance between acetone control and treatment. n, individual numbers used in significance test. nc, no choice, excluded in significance test. # or *, p < 0.05; ## or **, p < 0.01; ### or ***, p < 0.001; n.s., not significant. The same labels are used in following figures.

By using liquid chromatography (LC) analysis, we determined the hemolymph JH III titres with the developmental duration of both gregarious and solitarious fourth-instar nymphs. JH III titres of both phases showed similar dynamic patterns, increasing sharply at day 3 followed by a rapid decease (**Fig 1A**). However, JH III titres of solitarious nymphs displayed 2.0 and 2.9 times higher than that of gregarious nymphs at day 2 and day 3, respectively (**Fig 1A**). Consistent with the change of JH titres, the expression level of Krüppel homolog 1 (*Kr-h1*), a JH-response gene also increased rapidly from day 1 to day 3 and decreased sharply from day 3 to day 5 (**S1 Fig**). However, the expression level of JH receptor gene, *Methoprene-tolerant* (*Met*), did not change with the developmental duration of fourth-instar nymphs (**S1 Fig**).

To figure out whether JH has functional roles on locust attraction/repulsion behavior, we further carried out behavioral assay using Y-tube olfactometer (**Fig 1B**) after treated by the inhibitor of JH biosynthesis, precocene, or JH analog, methoprene, respectively. After treatment of precocene with different doses, 0 (acetone), 50, 100 and 150 μg (Ace, P50, P100 and P150) in gregarious nymphs, the survival rate decreased with increased doses and ranged from 100% in Ace-treated nymphs to 50% in P150-treated nymphs (**S2A Fig**). Most individuals after various doses treatments chose the ‘volatiles’ side, not differing with the controls (**S2B Fig**), whereas *Kr-h1* expression level significantly decreased in P100-treated gregarious nymphs (**S2C Fig**). After methoprene application, the survival rate of gregarious nymphs decreased with increased doses from 0 (acetone), 50, 75 to 100 μg (Ace, M50, M75 and M100), and ranged from 100% in Ace-treated nymphs to 85% in M100-treated nymphs (**S2D Fig**). M50-treatment sufficiently altered the choice behavior of gregarious nymphs from attraction to repulsion to gregarious volatiles (**S2E Fig**), and significantly enhanced *Kr-h1* expression level (**S2F Fig**). Therefore, we used 100 μg precocene and 50 μg methoprene for following pharmacological experiments.

We measured Y-tube performances of gregarious and solitarious nymphs at day 2 to day 4 after treatment by precocene or methoprene at day 1, respectively. Locust nymphs of both phases did not display significant behavioral change at day 2 (**Fig 1C–1F**). At both day 3 and 4, precocene treatment did not affect the choice behavior of gregarious nymphs (**Fig 1C**) whereas significantly decreased repulsion behavior of solitarious nymphs, that is 27% and 39% of treated solitarious nymphs chose the “air” side compared with 76% and 69% in the acetone control group, respectively (**Fig 1D**). Methoprene application induced significant behavioral change of gregarious nymphs from attraction to repulsion to gregarious volatiles (**Fig 1E**). We found that 74% and 68% of gregarious nymphs chose the “air” side compared with 24% and 31% of the acetone control group at both day 3 and 4, respectively (**Fig 1E**). Additional methoprene application on precocene-treated gregarious nymphs also significantly transited the behavior from attraction to repulsion to gregarious volatiles (**Fig 1G**). Solitarious nymphs treated by methoprene did not change their choice behavior at both day 3 and 4 (**Fig 1F**). However, additional methoprene application significantly rescued the repulsion behavior lost by precocene-treated solitarious nymphs (**Fig 1H**).

### The effects of *JHAMT* knockdown on attraction/repulsion behavior

**Fig 2:**
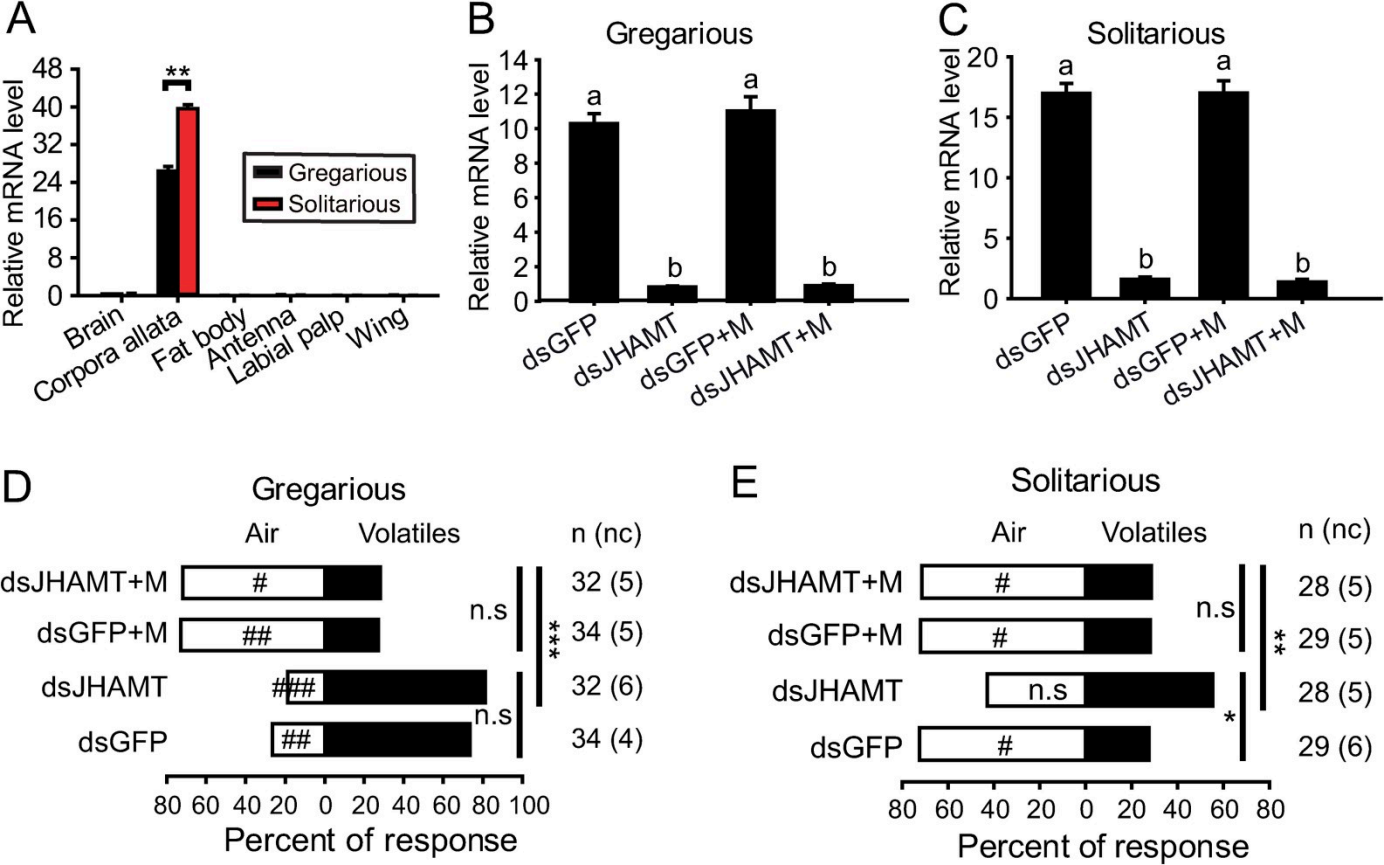
Effect of *JHAMT* knockdown on choice behavior in the fourth-instar nymphs. (A) Relative mRNA level of *JHAMT* in six selected tissues of gregarious and solitarious nymphs, respectively. (B) Relative mRNA level of *JHAMT* after injection of dsGFP or ds*JHAMT* or rescued by methoprene in gregarious nymphs. (C) Relative mRNA level of *JHAMT* after injection of ds*GFP* or ds*JHAMT* or rescued by methoprene in solitarious nymphs. (D) Choice behavior of gregarious nymphs at Day 3 after injection of ds*GFP* or ds*JHAMT* or rescued by methoprene in gregarious nymphs. (E) Choice behavior of gregarious nymphs at Day 3 after injection of ds*GFP* or ds*JHAMT* or rescued by methoprene in solitarious nymphs.

Juvenile hormone acid methyltransferase (*JHAMT*) has been demonstrated to catalyse the final or penultimate step of JH biosynthesis in various insect species **[25]**. We found that *JHAMT* expressed extremely high in corpora allata of both gregarious and solitarious fourth-instar nymphs at day 3 whereas displayed a 1.5-fold higher expression level in solitarious nymphs than that of gregarious nymphs (**Fig 2A**).

We then performed the RNAi-mediated knockdown of *JHAMT* in both gregarious and solitarious nymphs. Compared to the double-stranded *GFP* (ds*GFP*)-injected controls, ds*JHAMT* injection significantly suppressed *JHAMT* expression level in the corpora allata (**Fig 2B and 2C**). Additional methoprene application on ds*JHAMT*-injected nymphs did not increase *JHAMT* expression level (**Fig 2B and 2C**). Although *JHAMT* expression level decreased by 92.1%, we did not detect significant change in the response of gregarious nymphs to gregarious volatiles compared with ds*GFP*-injected controls. Methoprene application on either ds*GFP*- or ds*JHAMT*-injected gregarious nymphs significantly altered their behavior from attraction to repulsion to gregarious volatiles (**Fig 2D**). In solitarious nymphs, ds*JHAMT* injection significantly decreased *JHAMT* expression level to 9.4% of ds*GFP* controls (**Fig 2C**). The repulsion response of solitarious nymphs to gregarious volatiles was significantly altered (**Fig 2E**). Methoprene application on ds*JHAMT*-injected solitarious nymphs rescued their repulsion response to gregarious volatiles (**Fig 2E**).

### The effects of *Met* and *Kr-h1* knockdown on attraction/repulsion behavior

**Fig 3:**
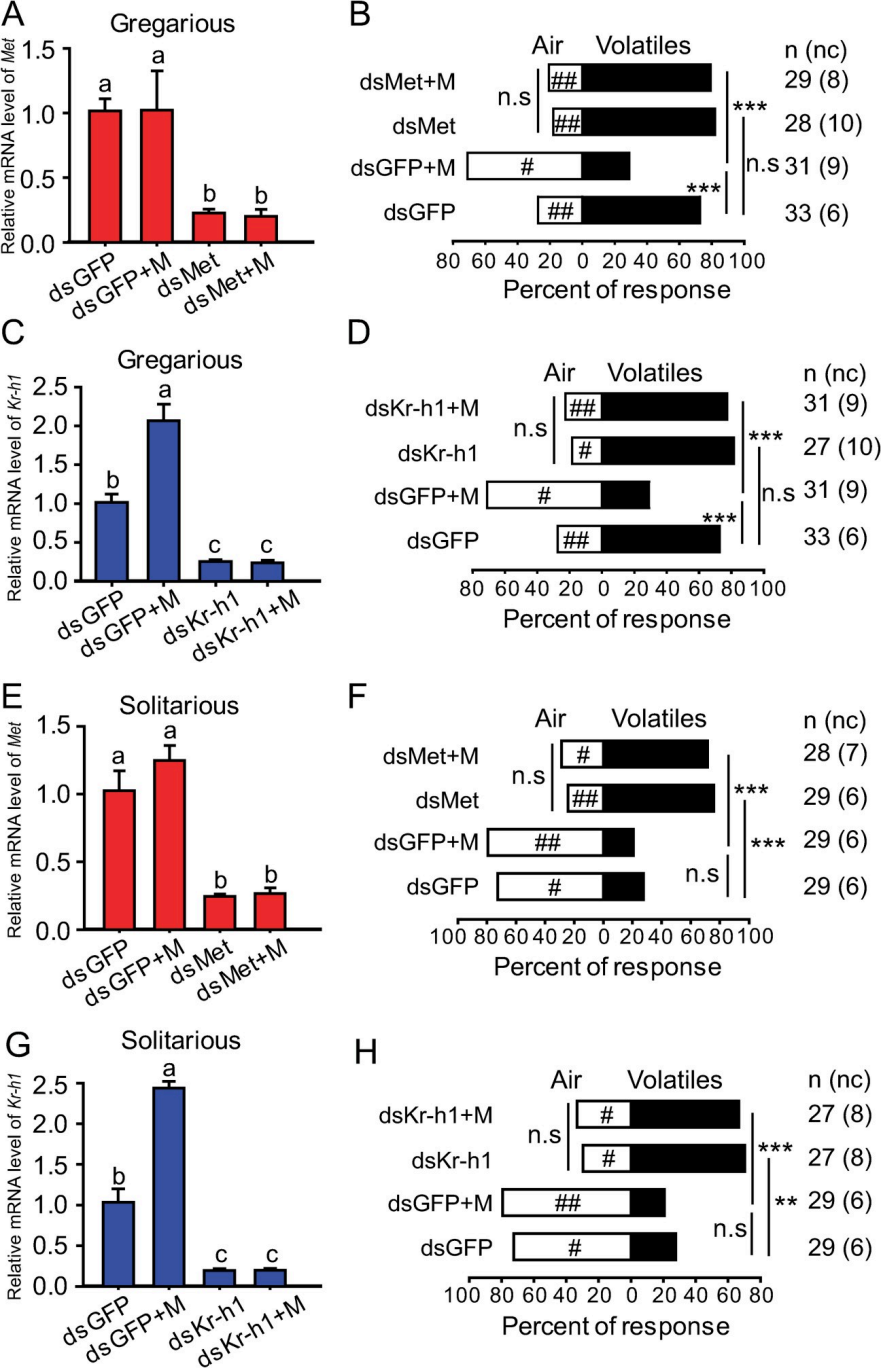
Effects of *Met* or *Kr-h1* RNAi on antennal gene expression and choice behavior in gregarious and solitarious nymphs. (A and B) Relative mRNA level of *Met* gene and choice behavior after injection of ds*GFP* or ds*Met* or rescued by methoprene in gregarious nymphs. (C and D) Relative mRNA level of *Kr-h1* gene and choice behavior after injection of ds*GFP* or ds*Kr-h1* or rescued by methoprene in gregarious nymphs. (E and F) Relative mRNA level of *Met* gene and choice behavior after injection of ds*GFP* or ds*Met* or rescued by methoprene in solitarious nymphs. (G and H) Relative mRNA level of *Kr-h1* gene and choice behavior after injection of ds*GFP* or ds*Kr-h1* or rescued by methoprene in solitarious nymphs.

To further investigate how behavioral effects of JH are mediated in locusts, we examined the behavioral responses after the knockdown of two key genes of JH signaling pathway, *Met* and *Kr-h1* in both gregarious and solitarious nymphs, respectively (**Fig 3**). Although the injection of ds*Met* or ds*Kr-h1* significantly decreased *Met* or *Kr-h1* expression level in antennal tissue to 22.2% and 24.8% of that in the ds*GFP* control, respectively (**Fig 3A and 3C**), the gregarious behavior was not affected by *Met* or *Kr-h1* knockdown (**Fig 3B and 3D**). However, the injection of ds*Met* or ds*Kr-h1* significantly blocked the repulsion responses of gregarious nymphs induced by methoprene application (**Fig 3B and 3D**). In solitarious nymphs, the injection of ds*Met* or ds*Kr-h1* significantly decreased *Met* or *Kr-h1* expression level in antennal tissue to 23.9% and 18.8% of that in the ds*GFP* control, respectively (**Fig 3E and 3G**). The solitarious behavior was significantly altered from repulsion to attraction after *Met* or *Kr-h1* knockdown (**Fig 3F and 3H**). Consistently, methoprene application did not induce the repulsion response of solitarious nymphs to gregarious volatiles after the injection of ds*Met* or ds*Kr-h1* (**Fig 3F and 3H**).

### JH-response transcriptomic profiles in antennal tissue

To reveal which genes are involved in JH-related olfactory plasticity in the peripheral olfactory system, RNA-seq were performed to identify the differentially expressed genes (DEGs) in antennal tissue between solitarious and gregarious nymphs (S/G) and between methoprene-treated and acetone-treated gregarious nymphs (M/A). Compared to gregarious nymphs, 445 genes were up-regulated and 545 genes were down-regulated in antennal tissue of solitarious nymphs. After gregarious nymphs were treated by methoprene, 74 genes were up-regulated and 48 genes were down-regulated in antennal tissue (S1 **Dataset**).

The DEGs in S/G and M/A were further categorized using several enrichment analyses including GO, KEGG and IPR, respectively. Shared enriched KEGG pathways were not identified in both S/G UP and M/A UP categories (S2 **Dataset**). However, we found that hemolymph juvenile hormone binding was the most enriched IPR item in both S/G UP and M/A UP categories (**Table 1**). This item included 10 DEGs that all belonged to takeout proteins in the S/G UP category and 5 DEGs that are takeout proteins and putative beta-carotene-binding proteins in the M/A UP category (**Table 2**). There were two shared takeout proteins, LOCMI15500 and LOCMI09174, in the S/G UP and M/A UP categories (**Table 2**).

**Table 1:** Top 5 enriched IPR categories in up-regulated genes of S/G and M/A datasets.

	IPR ID	IPR Title	Q-Value	Number of DEGs	Number of All Genes
**S/G UP**	**IPR010562**	**Haemolymph juvenile hormone binding**	**4.03E-04**	**10**	**50**
	IPR002401	Cytochrome P450, E-class, group I	4.03E-04	13	92
	IPR002213	UDP-glucuronosyl/UDP-glucosyltransferase	9.14E-04	11	73
	IPR004045	Glutathione S-transferase, N-terminal	2.65E-02	6	34
	IPR016160	Aldehyde dehydrogenase, cysteine active site	2.65E-02	4	13
**M/A UP**	**IPR010562**	**Haemolymph juvenile hormone binding**	**1.55E-04**	**5**	**50**
	IPR000618	Insect cuticle protein	1.42E-03	5	98
	IPR000172	Glucose-methanol-choline oxidoreductase	3.72E-03	3	27
	IPR011074	CRAL/TRIO, N-terminal domain	5.96E-03	3	34
	IPR013525	ABC-2 type transporter	2.71E-02	2	20

**Table 2:** Gene expression change of hemolymph juvenile hormone binding category.

	Gene ID	Fold Change	Protein Name
**S/G UP**	**LOCMI15500**	**17.0**	**TO1**
	LOCMI17117	10.5	Protein takeout
	LOCMI17054	8.6	Protein takeout
	LOCMI17164	5.8	Protein takeout
	LOCMI07354	5.4	Protein takeout
	LOCMI16576	4.5	Protein takeout
	LOCMI07227	3.6	Protein takeout
	LOCMI07724	3.3	Protein takeout
	LOCMI09172	3.0	Protein takeout
	**LOCMI09174**	**2.5**	**Protein takeout**
**M/A UP**	LOCMI14013	226.6	Putative beta-carotene-binding protein
	LOCMI14014	19.5	Putative beta-carotene-binding protein
	**LOCMI15500**	**2.2**	**TO1**
	**LOCMI09174**	**2.1**	**Protein takeout**
	LOCMI09998	1.7	Putative beta-carotene-binding protein

**Fig 4:**
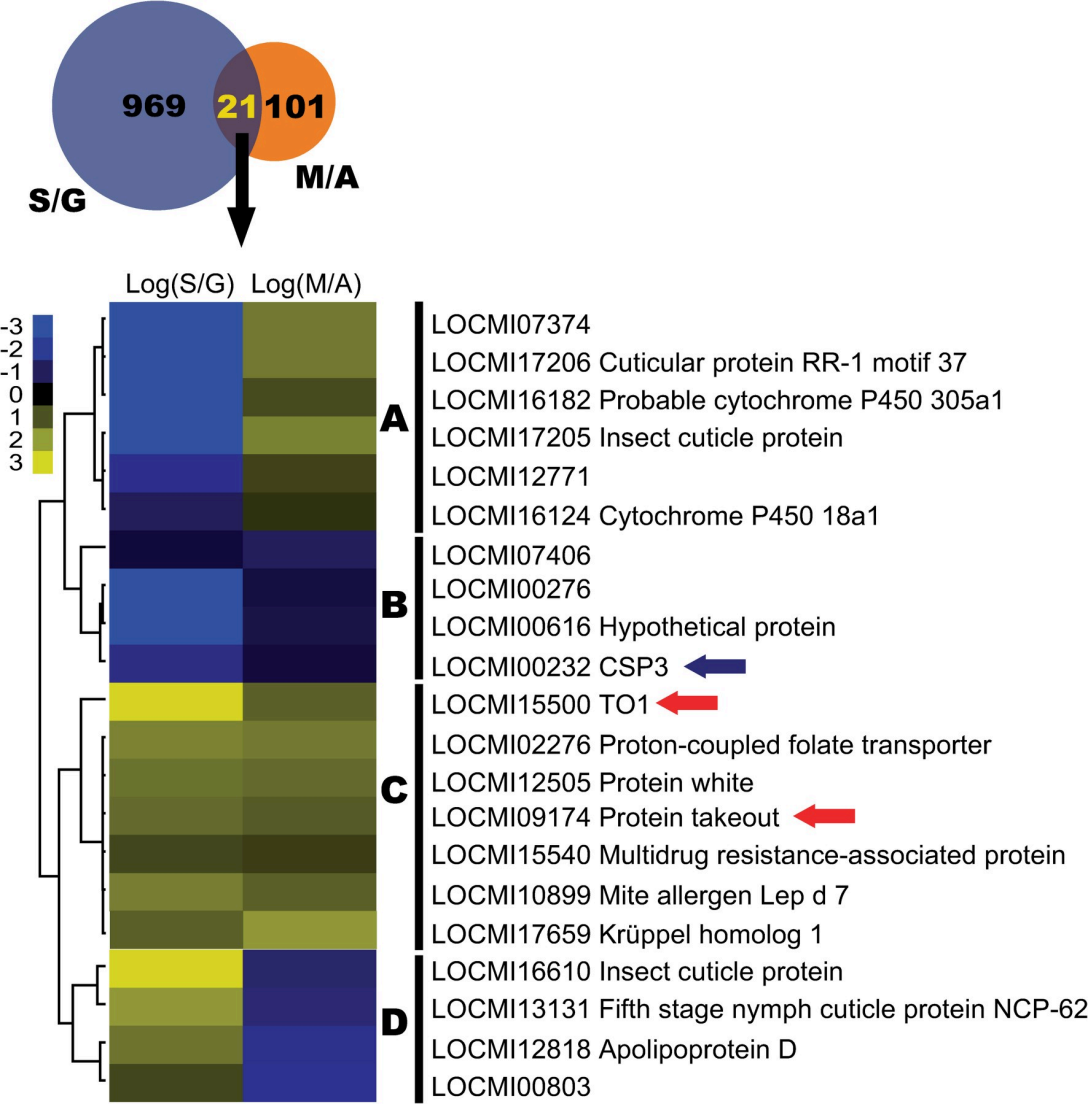
Cluster analysis of 21 mutual DEGs between S/G and M/A. DEGs, differentially expressed genes; Log, the logarithm of fold change to base 2. G, gregarious; S, solitarious; M, methoprene-treated gregarious; A, acetone-treated gregarious control. Red arrow, *takeout* gene; blue arrow, *CSP* gene.

A cross analysis was further performed between DEGs from S/G and M/A datasets. We identified 21 mutual DEGs that mainly included insect cuticle protein, cytochrome P450 and olfactory-related genes. These genes were further categorized into four clusters (**Fig 4**). Olfactory genes LOCMI00232, and LOCMI15500 and LOCMI09174 were categorized into cluster B and cluster C, respectively. LOCMI00232 displayed reverse gene expression pattern with both LOCMI15500 and LOCMI09174. Interestingly, LOCMI00232 and LOCMI15500 were *CSP3* and *TO1* genes, respectively, which have been reported to play regulatory roles in attraction/repulsion behavior in our previous work **[15]**. We also found that the *Kr-h1* expression level was also increased by methoprene application on gregarious nymphs (**Fig 4**, S1 **Dataset**).

### The effects of JH signaling on *TO1* and *CSP3* gene expression and choice behavior

**Fig 5:**
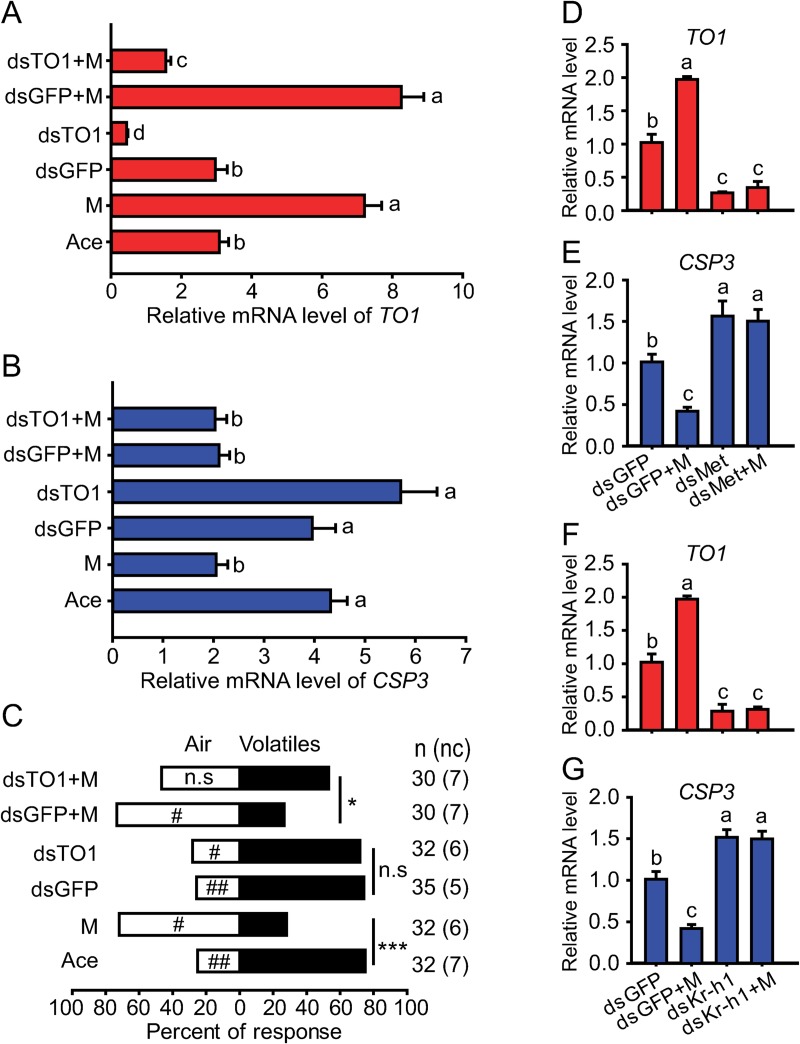
Effects of JH or JH signaling pathway genes on antennal gene expression and choice behavior in gregarious nymphs. (A) Relative mRNA level of *TO1* gene after methoprene treatment or injection of ds*GFP* or ds*TO1* combined with methoprene treatment. (B) Relative mRNA level of *CSP3* gene after methoprene treatment or injection of ds*GFP* or ds*TO1* combined with methoprene treatment. (C) Choice behavior after methoprene treatment or injection of ds*GFP* or ds*TO1* combined with methoprene treatment. (D and E) Relative mRNA level of *TO1* or *CSP3* genes after injection of ds*GFP* or ds*Met* or rescued by methoprene. (F and G) Relative mRNA level of *TO1* or *CSP3* genes after injection of ds*GFP* or ds*Kr-h1* or rescued by methoprene. M, methoprene.

We investigated whether JH signaling pathway modulates olfactory behavior *via* regulating *TO1* and *CSP3* genes. After methoprene treatment, the *TO1* expression level increased by 2.4-fold and *CSP3* decreased to 49.8% of the acetone controls in gregarious nymphs (**Fig 5A and 5B**). Methoprene application on gregarious nymphs reversed the behavioral response from attraction to repulsion to gregarious volatiles in contrast to the acetone controls (**Fig 5C**). ds*TO1* injection significantly decreased the *TO1* expression level to 14.3% of the ds*GFP*-injected control while no behavioral effect on gregarious nymphs (**Fig 5A and 5C**). After combination of the ds*TO1* injection with methoprene treatment, the behavioral effect of methoprene significantly declined (**Fig 5C**). Meanwhile, *CSP3* expression level was exclusively down-regulated when methoprene treatment was performed (**Fig 5B**).

We further found that *Met* or *Kr-h1* knockdown also significantly reduced *TO1* expression level (**Fig 5D and 5F**) and enhanced *CSP3* expression level (**Fig 5E and 5G**). The effects of methoprene on *TO1* and *CSP3* expression was significantly blocked by ds*Met* and ds*Kr-h1* injection (**Fig 5D–5G**). According to previous report **[26]**, we have also predicted several *Kr-h1* binding sites on the probable promoter regions of both *TO1* and *CSP3* (**S3 and S4 Figs**). Thus, these results indicated that *TO1* and *CSP3* are probably the downstream genes of the *Kr-h1*.

## Discussion

Locust phase polyphenism provides an excellent model with which to understand the regulatory roles of hormones in animal behavior **[7, 8]**. It is well known for the effects of JH on several phase characteristics of locusts, including body colour, pheromone production and oocyte maturation **[6, 7]**, however its roles in behavioral changes remain a long-standing debate. Here, we provide evidence for the roles of juvenile hormone in olfactory-mediated behavioral plasticity by suppressing locust attraction behavior. We then demonstrated that the behavioral effect of JH signaling pathway is mediated through modulating expression levels of two olfactory genes *TO1* and *CSP3* that are involved in locust phase change. Our results provided novel molecular mechanisms underlying the roles of juvenile hormone in insect behavioral plasticity.

Our results demonstrated functional roles of JH in phase-related olfactory-mediated behavior of *L*. *migratoria* nymphs. The possible effects of JH on locust aggregation behavior have been implied in previous studies **[24, 27]**; however, the detailed mechanisms were not investigated. Here we revealed the regulatory mechanism of attraction/repulsion behavior by linking the JH titres, JH pathway genes and olfactory genes in the migratory locust. JH deprivation and *JHAMT* knockdown experiments together suggested higher JH titres can trigger avoidance response to conspecific volatiles in solitarious nymphs. Meanwhile, JH application in gregarious nymphs led to significantly suppressive effects on attraction behavior. These results provided enough evidence that JH is involved in olfactory-mediated behavioral plasticity. In several other insects, JH action on olfactory-mediated behavior has also been reported, for example, the host-seeking behavior mediated by the reception of odours from hosts in *Culex pipiens* **[28]**, the increase in behavioral responsiveness towards the female sex pheromone in the male moth, *Agrotis ipsilon* **[29]**, the shift of foraging behavior with age in the honeybee, *Apis mellifera* **[30]**, and the enhancement of male courtship success in *Drosophila melanogaster* **[31]**, indicating that conserved roles of JH in olfactory-mediated behavioral plasticity in variant insect species.

The analysis of antennal transcriptome showed the significant effect of JH on gene expression of the antennae in gregarious nymphs. Interestingly, two of the JH-response genes, *TO1 and CSP3*, have been demonstrated to mediate the shift between attraction and repulsion behavior in the migratory locust **[15]**. The JH analog treatment increased the expression levels of *TO1* but suppressed the expression levels of *CSP3*. The effect of JH on expression patterns of these two genes is consistent with its effect on behavioral changes in gregarious locusts. In addition, the expression of several key genes involved in insect hormone synthesis pathway in the antennae have been influenced by JH analog application, for example, *juvenile hormone esterase* **[32]** and *cytochrome P450 18a1* **[33]**, indicating a possible interaction between JH and 20-hydroxyecdysone in regulating olfactory-mediated behavioral plasticity. Surprisingly, JH-response genes include few olfactory receptor (OR) genes, which are key molecular components involved in peripheral odour detection. This result implied that OR genes could not be the downstream targets of JH signaling pathway. Certainly, we cannot exclude that the differential expression of OR genes might be involved in phase-related olfactory-evoked plasticity in locusts. A number of studies have reported that the differences of the antennal OR genes expression mediate behavioral changes depending on nutritional states, age and social experience **[34]**.

Our data demonstrated that *TO1* and *CSP3* are necessarily required for the effects of JH signaling on locust aggregation behavior. The RNAi of *TO1* significantly reduced JH-induced repulsive response in the gregarious locusts, consistent with the behavioral role of *TO1* in locust phase change **[15]**. Knockdown of JH receptor, *Met*, or a downstream transcription factor, *Kr-hl*, can also affect the expression levels of *TO1* and *CSP3* and behavioral responses. In addition, both *Met* and *Kr-h1* knockdown can block the JH action on the expression of *TO1* and *CSP3*. These results indicated that behavioral effect of JH is through simultaneously regulating the opposite expression of *TO1* and *CSP3* via the *Met-Kr-h1* signaling pathway. The regulatory effects of JH on *takeout* genes have also been reported in other insect species, including the brown plant hopper, *Nilaparvata lugens* **[35],** the tobacco hornworm, *Manduca sexta* **[36]**, the termite, *Reticulitermes flavipe* **[37]** and the honeybee *Apis mellifera* **[38]**. *takeout* genes are even regarded as a JHBP superfamily member and recently have been found to be specifically expressed in chemosensory organs, such as antennae and labella, suggesting their functions in chemical perception in insects **[15, 39, 40]**. It seems that JH action on *takeout* genes is also governed by the JH/*Met-Kr-h1* signaling pathway, which plays important roles in development, metamorphosis and reproduction **[41–43]**. However, to our knowledge, our results represent the first report of the JH signaling pathway regulating the chemosensory protein, *CSP3*. For the detailed mechanisms underlying how JH/Met-Kr-h1 simultaneously regulates the opposite expression of *CSP3* and *TO1*, we hypothesized: 1) *Kr-h1* acts as the downstream transcription factors of *Met* to directly bind to the promoters of these two genes, as a transcriptional activator of *TO1* and a transcriptional repressor of *CSP3*, which has been proposed in mosquitoes **[26]** or 2) *Kr-h1* might initiate the expression of other transcriptional factors which regulate the expression of *TO1* and *CSP3*. In fact, we have predicted several *Kr-h1* binding sites on probable promoter regions of these two genes (**S3 and S4 Figs**) based on the locust genome data **[44]** and previous report **[26]**, implying possible direct binding of *Kr-h1* to promoter motifs of *TO1* and *CSP3*. Thus, further work needs to be done to reveal the detailed mechanisms underlying the regulation of *Kr-h1* on the expression of these two genes.

The results from the current study indicate that JH action on aggregation behavior is through influencing the peripheral nervous system, as *TO1* and *CSP3* are mainly expressed in antennal tissue **[15]**. Actually, the influences of JH on peripheral neuronal plasticity have also been reported in other insect species **[3, 45, 46].** A recent study has further demonstrated that the effect of JH on age-dependent sensitivity of Or47b neuron can coordinate male courtship behavior in *Drosophila melanogaster* **[31]**. Our results provide more detailed molecular mechanisms underlying the JH control of olfactory-mediated behavioral plasticity through influencing peripheral neuronal plasticity. In fact, JH is essential for the development and organization of neural circuits including the peripheral and central nervous systems of insects **[47, 48]**. In the desert locust *S*. *gregaria*, JH can activate the responsiveness of olfactory interneurons in the antennal lobe **[27]**. Our previous studies have suggested that phase-related behavioral plasticity involves multiple-level mechanisms including the peripheral and central nervous systems **[6]**. Several biogenic amines such as dopamine, tyramine, octopamine and serotonin, have been revealed to play important roles in the process of locust behavioral changes **[17, 49, 50]**. JH may act as a coordinator of phase-related aggregation behavior in response to population density by modulating the peripheral and central olfactory neuronal sensitivity in the migratory locust. Future investigation into the mechanisms underlying pleiotropy should lead to a deeper understanding of the ways by which JH mediates phase-related behavioral plasticity in response to the changes of population density.

In summary, this study indicates that juvenile hormone acts on the expression of olfactory-related genes in peripheral chemosensory organs to change aggregation behavior in locusts. Our finding of a mechanism by which sensory detection is modified by a hormone provides new insights into the precise modulation of phenotypic plasticity in the animal kingdom.

## Materials and methods

### Experimental insects

The migratory locust, *L*. *migratoria*, used in this study were from colonies maintained at the Institute of Zoology, Chinese Academy of Sciences. Gregarious cultures were reared in large, well-ventilated cages (25 cm × 25 cm × 25 cm) at densities of 200–300 insects per container. Solitarious cultures were reared in isolation in separate ventilated cages (10 cm × 10 cm × 25 cm) with charcoal-filtered compressed air. This method retained the phase-traits for reversible phase transition due to physical, visual and olfactory isolation. Both colonies were reared under a 14:10 light/dark photo regime at 30 ± 2°C and on a diet of fresh, greenhouse-grown wheat seedlings and wheat bran. Fourth-instar gregarious or solitarious nymphs were used in all of the following experiments. Most fourth-instar gregarious or solitarious nymphs developed for 5 days before moulting to fifth-instar. For convenience of description, we defined Day 1 (D1) as within 12 hours post moult from third-instar, and Day 2 to Day 5 (D2-D5) as the second to fifth day post moult from third-instar.

### Quantification of hemolymph JH III

Hemolymph from 4 individuals of gregarious or solitarious nymphs was pooled together as one biological replicate and 8 biological replicates were sampled at D1, D2, D3, D4 and D5, respectively. The procedure was as previously described **[51]**. Briefly, 40 μl hemolymph was mixed with 100 μl 70% methanol and 200 μl hexane, followed by centrifugation at 4,500 ×g for 10 min. The upper hexane layer was then dried with nitrogen, dissolved in 50% methanol and sonicated for 10 min. After centrifugation twice at 14,000 ×g for 10 min, the supernatant was collected for liquid chromatography (LC) analysis using Nexera UHPLC LC-30A (Shimadzu) equipped with two LC-30AD pumps, a SIL-30AC autosampler, a CTO-30A thermostated column compartment and a DGU-20A5 degasser. An ACQUITY UPLC BEH C18 column was used for LC separation. Mass spectrometric detection (MS/MS/MS) was then carried out using AB SCIEX Triple Quad 4500 (Applied Biosystems) with an electrospray ionization source (Turbo Ionspray) in a positive electrospray ionization mode. Three major JH III specific ions [m/z = 235.3, m/z = 189.0, and m/z = 147.0] were monitored. Data acquisition and processing were performed using AB SCIEX Analyst 1.6 software (Applied Biosystems).

### Precocene and methoprene application

In dose experiments, within 12 h post moult from third-instar, the fourth-instar gregarious and solitarious nymphs were treated with ethoxyprecocene (50 μg, 100 μg and 150 μg dissolved in 2 μl of acetone, respectively. Sigma-Aldrich Ltd.) or s-(+)-methoprene, a JH analog (50 μg, 75 μg and 100 μg dissolved in 2 μl of acetone, respectively. Santa Cruz Biotech) by topical application. Control locusts were treated with 2 μl of acetone. In all other following experiments, 100 μg ethoxyprecocene or 50 μg s-(+)-methoprene was topically applied to individual fourth-instar nymphs. In JH rescue experiments, locust nymphs were treated by methoprene after 12 h by prococene treatment.

### Behavioral assay

The behavioral assay method was as previously described **[15]**. Briefly, a Y-tube olfactometer was used to analyze the behavioral responses of individual locust to volatiles from 30 fourth-instar gregarious nymphs in the absence of any visual cues. Individual nymph was recorded as “first choice” for volatile or air (whenever the locust moved more than 5 cm into either arm) or “no choice” (n.c) in 5 minutes.

### Total RNA isolation and qRT-PCR

Total RNA from antennae, labial palps, wings, brains, corpora allata and fat body tissues of gregarious and solitarious nymphs was extracted using Trizol reagent (Invitrogen) according to the manufacturer’s protocol. The cDNA was reverse transcribed from 2 μg of total RNA using MMLV reverse transcriptase (Promega). Quantitative real-time PCR (qRT-PCR) was conducted using a LightCycler480 (Roche) and RealMasterMix (SYBR Green) kit (Tiangen), initiated with a 10 min incubation at 95°C, followed by 45 cycles at 95°C, 10 s at 58°C, and 20 s at 72°C. A melting curve analysis was performed to confirm the specificity of amplification. The gene ribosomal protein 49 (*Rp49*) was chosen as a reference for normalizing the mRNA levels. Primer sequences were listed in **S1 Table**. The relative quantity method was used to measure the relative mRNA expression level. Four biological replicates were performed for each treatment.

### RNAi

Double-stranded RNA (dsRNA) of *GFP* (green fluorescent protein), *JHAMT* (*juvenile hormone acid methyltransferase*, GenBank: AXM43874), *TO1* (*takeout 1*, GenBank: GU722575), *Met* (*methoprene-tolerant*, GenBank: KF471131) and *Kr-h1* (*krüppel homolog 1*, GenBank: KJ425482) was prepared using T7 RiboMAX^TM^ Express RNAi system (Promega) following the manufacturer’s instructions. The primer sequences for double-stranded RNA synthesis were listed in **S1 Table**. Fourth-instar nymphs within 12 h post moult were injected with 18 μg (6 μg/μl) of double-stranded RNA of *GFP* (ds*GFP*) or double-stranded RNA of the target genes in the second ventral segment of the abdomen. In the RNAi and methoprene double-treatment experiment, gregarious nymphs were treated by methoprene after 12 h by dsRNA injection. At day 3, choice behavior of individuals was assessed, and corpora allata or antennal tissue were sampled for qRT-PCR analysis.

### RNA-seq and bioinformatic analysis

The RNA-seq of methoprene- or acetone-treated gregarious antennae and gregarious or solitarious antennae was performed using a HiSeq 2500 platform (BGI Shenzhen). Three biological replicates were performed for each treatment. To calculate the gene expression, the RNA-seq reads were mapped to the locust genome reference **[44]** using Tophat2 (version 2.0.13). The unique reads number that mapped to every gene model was counted using HTseq. The locust gene model GFF file was produced from locust genome project. EdgeR package was used to detect the differentially expressed genes, which were defined as with fold change > 1.5 and p < 0.05. Enrichment analysis for the supplied gene list was carried out based on an algorithm presented by GOstat with the whole annotated gene set as the background **[44, 52]**. The p value was approximated using the Chi-square test. Fisher’s exact test was used when any expected value of count was below 5. For the GO and IPR enrichment analyses, in order to obtain succinct results, if one item was ancestor of another and the enriched gene list of these two items were the same, the ancestor item was deleted from the results.

### Statistical analysis

Statistical analyses were performed using Student’s t-test or one-way analysis of variance (ANOVA) followed by a Tukey’s test for multiple comparisons. Significant difference was considered at p < 0.05. Values were reported as mean ± SE. Behavior assay data were analysed by Mann-Whitney U test because of its non-normal distribution characteristics. Chi square test was used for significance test between numbers of nymphs in each arm of Y-tube olfactometer. Differences were considered significant at p < 0.05. Data were analysed using IBM SPSS Statistics v.19 software (SPSS Inc). All the data underlying the main figures were deposited in S3 Dataset.

## Supporting information

S1 Table Primers used in qRT-PCR and dsRNA synthesis. (DOCX)

S1 Fig Expression of JH signaling pathway genes during development of gregarious and solitarious fourth-instar nymphs. (A) Relative mRNA level of *Kr-h1* gene in antennae of gregarious nymphs. (B) Relative mRNA level of *Met* gene in antennae of solitarious nymphs. D1, within 12 hours post moult from third instar. D2-D5, the second to fifth day post moult from third instar. Student’s t-test was used for significance test of gene expression level between gregarious and solitarious nymphs. *, p < 0.05.
(TIF)

S2 Fig Dose responses of fourth-instar gregarious nymphs to precocene or methoprene. (A) Survival rate after precocene treatment. (B) Choice behavior after precocene treatment. (C) Relative mRNA level of *Kr-h1* gene in antennae after precocene treatment. (D) Survival rate after methoprene treatment. (E) Choice behavior after methoprene treatment. (F) Relative mRNA level of *Kr-h1* gene in antennae after methoprene treatment. Ace, acetone; P, precocene; M, methoprene. Numbers following P or M mean treatment dose (μg). Volatiles represent volatiles emanating from 30 fourth-instar gregarious nymphs. Significance marks inside white stripe indicate the significance between individuals choosing air side and volatiles side. Marks outside the stripe indicate the significance between acetone control and treatment. n, individual numbers used in significance test. nc, no choice, excluded in significance test. *, p < 0.05; **, p < 0.01; ***, p < 0.001; n.s., not significant.
(TIF)

S3 Fig Predicted *Kr-h1* binding sites (KBS) in the 3000 bp upstream promoter sequence of *TO1* gene. Six sense and 10 antisense (-) KBS sequences were identified on position 1313, 1370, 2135, 2507, 2560, 2653, -448, -488, -1026, -1059, -1199, -1248, -1896, -2121, -2150 and -2658 according to KBS core sequence (12 bp): [GAT][AG][CGAT][CAT][TA][ATG][CTG][CGT][CGTA][CAG]AA. Sense KBS sequences are in red and antisense KBS sequences are in blue. The merged sequences between the sense and antisense were underlined.
(TIF)

S4 Fig Predicted *Kr-h1* binding sites (KBS) in the 3000 bp upstream promoter sequence of *CSP3* gene. Fifteen sense and 9 antisense (-) KBS sequences were identified on position 87, 199, 533, 750, 802, 1018, 1279, 1441, 1626, 1757, 2177, 2420, 2702, 2746, 2969, -165, -999, -1191, -2143, -2162, -2407, -2428, -2833 and -2901. Sense KBS sequences are in red and antisense KBS sequences are in blue. The merged sequences between the sense and antisense were underlined.
(TIF)

S1 Dataset Differentially expressed genes (DEGs) between gregarious and solitarious antennae (S/G), and between methoprene-treated and acetone-treated antenna (M/A). (XLSX)

S2 Dataset Enriched Gene Ontology categories, KEGG pathways and IPR items. (XLSX)

S3 Dataset All numerical data underlying the main figures. (XLSX)
