# Supplementary material for: Juvenile hormone suppresses aggregation behavior through influencing antennal gene expression in locusts
Source: PLoS Genet. 2020 Apr 29;16(4):e1008762. doi: 10.1371/journal.pgen.1008762 (PMC7213744; doi:10.1371/journal.pgen.1008762)
Supplement: S1 Table — (DOCX) [file pgen.1008762.s001.docx]

Table S1. Primers used in qRT-PCR and dsRNA synthesis.

| Name | Forward | Reverse |  |
| --- | --- | --- | --- |
| qRT-PCR | | | |
| *Rp49* | CGTAAACCGAAGGGAATTGA | GAAGAAACTGCATGGGCAAT | |
| *JHAMT* | AACAACAACAACAAGAGCGGAA | CGTTCGGATCTCCATCGTGTC | |
| *TO1* | GGAAGTTCTGAGCGACTACACGG | CGAACGTCAGGATTATTGGGAC | |
| *CSP3* | CCTCGTCAACAACAAGCCC | CCAAACACTCATGCAACAC | |
| *Met* | GTGCCTGAAGAAGAAGAAC | GGAGGTGATGAAGGAGAG | |
| *Kr-h1* | ACTTCGTCTTCTGGAATGA | GGCAATCGGTATTACACTTAG | |
| dsRNA synthesis | | | |
| *GFP* | CACAAGTTCAGCGTGTCCG | GTTCACCTTGATGCCGTTC | |
| *JHAMT* | TGCGAATCTACACCGACTGC | TCCACCGACACGAGCTTCT | |
| *TO1* | ATGAAGAAGAACGACGGGA | TGAGGAGTGGTGGAGGCTA | |
| *Met* | TTAGGGCAGCATCAGAAAG | TCGTCGGGAGGAAGTGTAT | |
| *Kr-h1* | GTCAAGGAGAACCTGAGCGTGC | TGCTGCTGCTCCGAGTGGCT | |
