# Supplementary figures and images for: Juvenile hormone suppresses aggregation behavior through influencing antennal gene expression in locusts
Source: PLoS Genet. 2020 Apr 29;16(4):e1008762. doi: 10.1371/journal.pgen.1008762 (PMC7213744; doi:10.1371/journal.pgen.1008762)

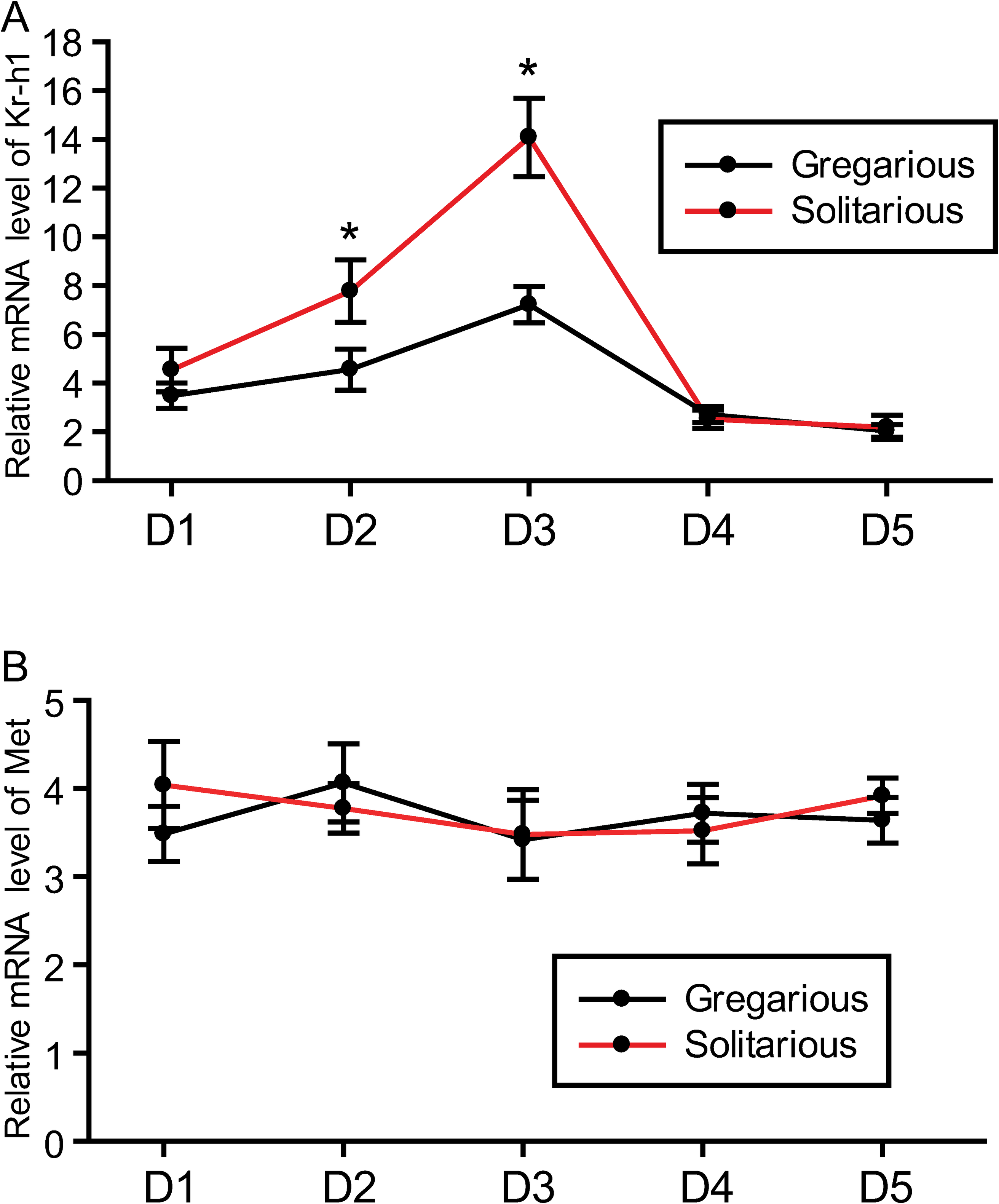

Supplement: S1 Fig — (A) Relative mRNA level of Kr-h1 gene in antennae of gregarious nymphs. (B) Relative mRNA level of Met gene in antennae of solitarious nymphs. D1, within 12 hours post moult from third instar. D2-D5, the second to fifth day post moult from third instar. Student’s t-test was used for significance test of gene expression level between gregarious and solitarious nymphs. *, p < 0.05. (TIF) [file pgen.1008762.s002.tif]

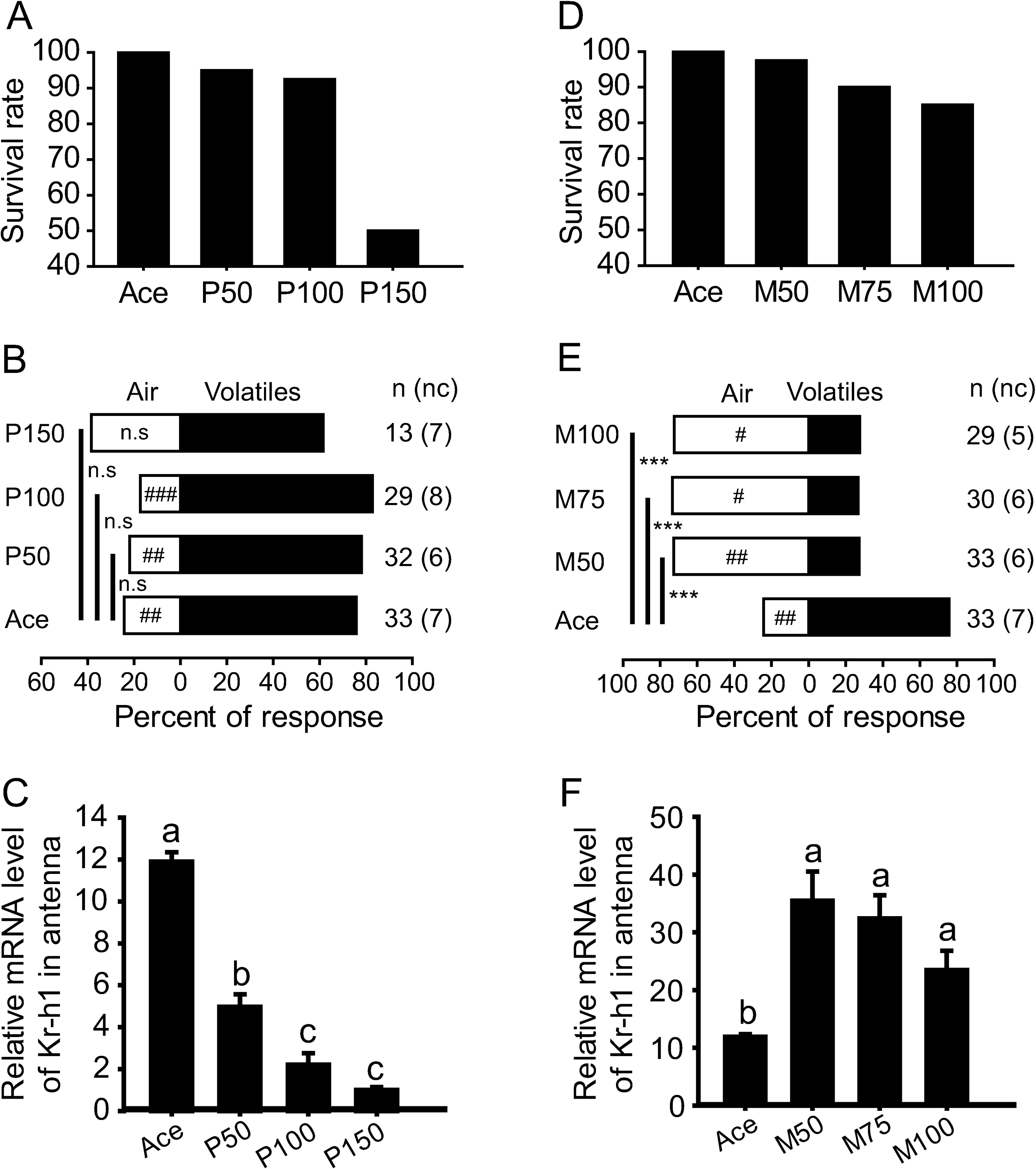

Supplement: S2 Fig — (A) Survival rate after precocene treatment. (B) Choice behavior after precocene treatment. (C) Relative mRNA level of Kr-h1 gene in antennae after precocene treatment. (D) Survival rate after methoprene treatment. (E) Choice behavior after methoprene treatment. (F) Relative mRNA level of Kr-h1 gene in antennae after methoprene treatment. Ace, acetone; P, precocene; M, methoprene. Numbers following P or M mean treatment dose (μg). Volatiles represent volatiles emanating from 30 fourth-instar gregarious nymphs. Significance marks inside white stripe indicate the significance between individuals choosing air side and volatiles side. Marks outside the stripe indicate the significance between acetone control and treatment. n, individual numbers used in significance test. nc, no choice, excluded in significance test. *, p < 0.05; **, p < 0.01; ***, p < 0.001; n.s., not significant. (TIF) [file pgen.1008762.s003.tif]

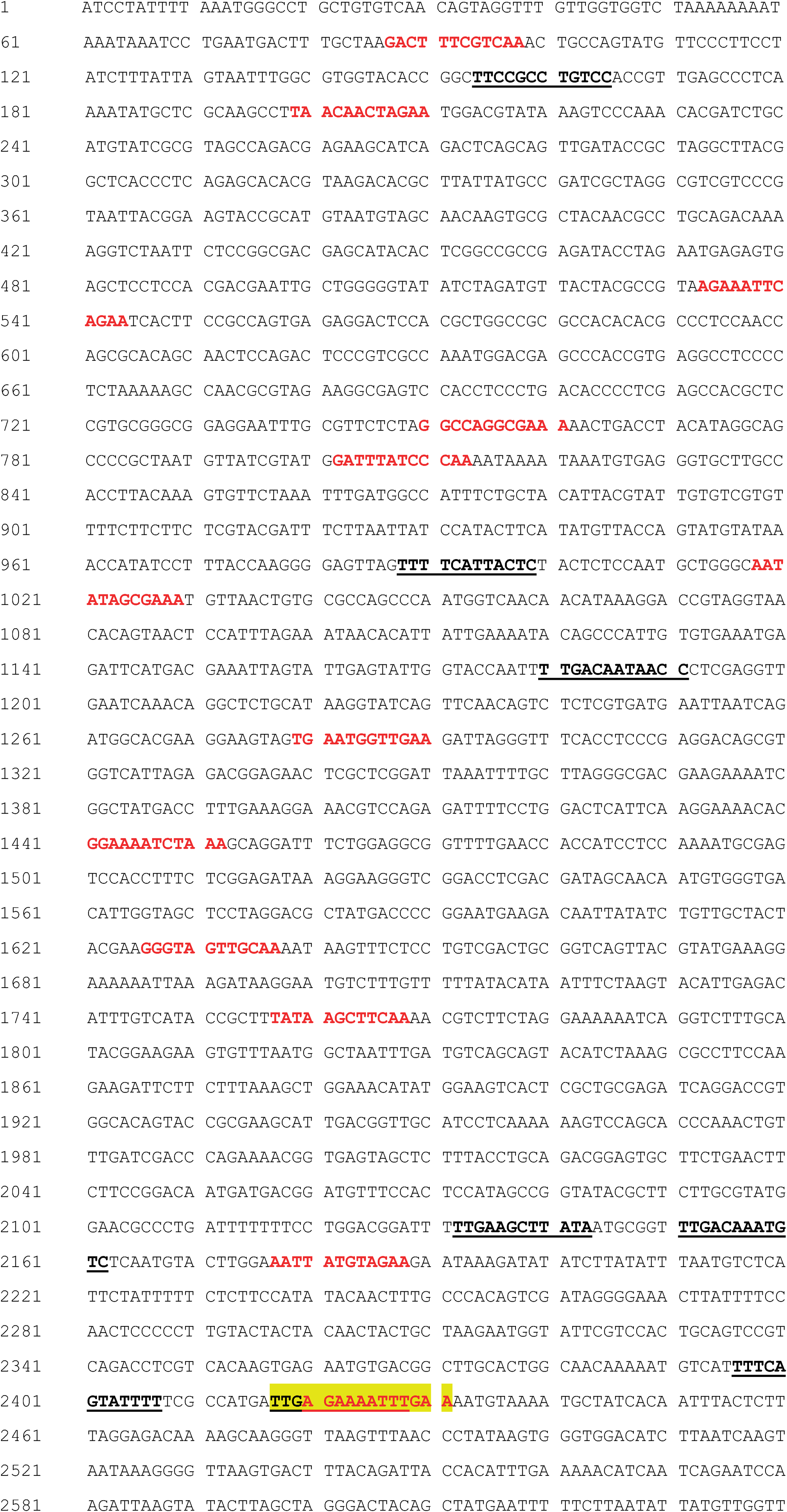

Supplement: S3 Fig — Six sense and 10 antisense (-) KBS sequences were identified on position 1313, 1370, 2135, 2507, 2560, 2653, -448, -488, -1026, -1059, -1199, -1248, -1896, -2121, -2150 and -2658 according to KBS core sequence (12 bp): [GAT][AG][CGAT][CAT][TA][ATG][CTG][CGT][CGTA][CAG]AA. Sense KBS sequences are in red and antisense KBS sequences are in blue. The merged sequences between the sense and antisense were underlined. (TIF) [file pgen.1008762.s004.tif]

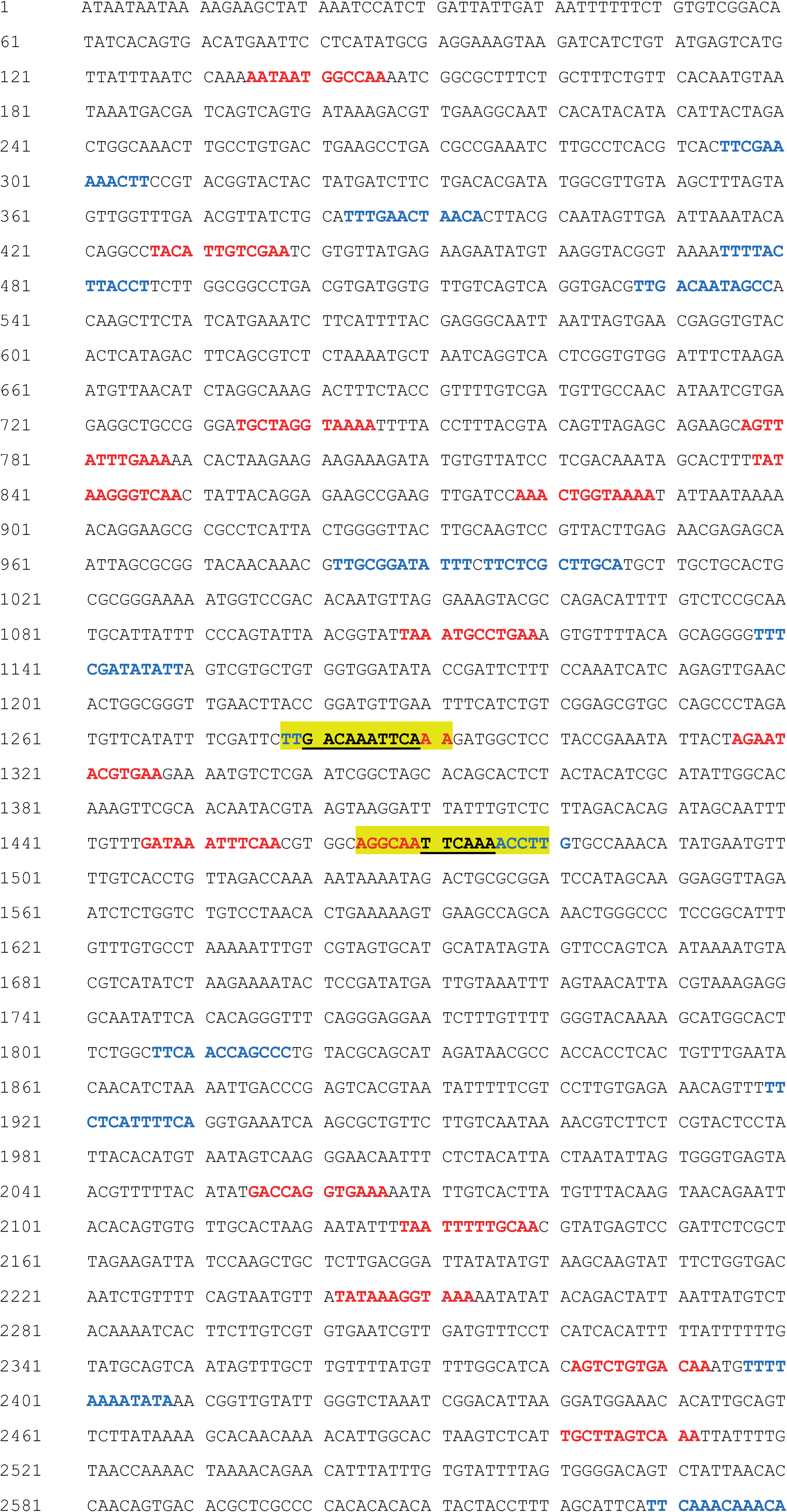

Supplement: S4 Fig — Fifteen sense and 9 antisense (-) KBS sequences were identified on position 87, 199, 533, 750, 802, 1018, 1279, 1441, 1626, 1757, 2177, 2420, 2702, 2746, 2969, -165, -999, -1191, -2143, -2162, -2407, -2428, -2833 and -2901. Sense KBS sequences are in red and antisense KBS sequences are in blue. The merged sequences between the sense and antisense were underlined. (TIF) [file pgen.1008762.s005.tif]
